# The role of phospholipase C signaling in bovine herpesvirus 1 infection

**DOI:** 10.1186/s13567-017-0450-5

**Published:** 2017-09-07

**Authors:** Liqian Zhu, Chen Yuan, Xiuyan Ding, Clinton Jones, Guoqiang Zhu

**Affiliations:** 1 College of Veterinary Medicine, Yangzhou University, 48 Wenhui East Road, Yangzhou, 225009 Jiangsu China; 2 Jiangsu Co-innovation Center for Prevention and Control of Important Animal Infectious Diseases and Zoonoses, 48 Wenhui East Road, Yangzhou, 225009 Jiangsu China; 3 Test Center, Yangzhou University, 48 Wenhui East Road, Yangzhou, 225009 Jiangsu China; 4 Department of Veterinary Pathobiology, Oklahoma State University, Center for Veterinary Health Sciences, Stillwater, OK 74078 USA

## Abstract

Bovine herpesvirus 1 (BoHV-1) infection enhanced the generation of inflammatory mediator reactive oxidative species (ROS) and stimulated MAPK signaling that are highly possibly related to virus induced inflammation. In this study, for the first time we show that BoHV-1 infection manipulated phospholipase C (PLC) signaling, as demonstrated by the activation of PLCγ-1 at both early stages [at 0.5 h post-infection (hpi)] and late stages (4–12 hpi) during the virus infection of MDBK cells. Viral entry, and de novo protein expression and/or DNA replication were potentially responsible for the activation of PLCγ-1 signaling. PLC signaling inhibitors of both U73122 and edelfosine significantly inhibited BoHV-1 replication in both bovine kidney cells (MDBK) and rabbit skin cells (RS-1) in a dose-dependent manner by affecting the virus entry stage(s). In addition, the activation of Erk1/2 and p38MAPK signaling, and the enhanced generation of ROS by BoHV-1 infection were obviously ameliorated by chemical inhibition of PLC signaling, implying the requirement of PLC signaling in ROS production and these MAPK pathway activation. These results suggest that the activation of PLC signaling is a potential pathogenic mechanism for BoHV-1 infection.

## Introduction

Bovine Herpesvirus 1 (BoHV-1) is an enveloped virus belonging to *Alphaherpesvirinae* subfamily member [1]. Acute infection of cattle with BoHV-1 generally results in inflammatory disease within the upper respiratory tract, nasal cavity, and ocular cavity, which leads to erosion of the mucosal surface. BoHV-1 also suppresses host immune responses by diverse mechanisms which lead to secondary infections [2]. BoHV-1 together with other viruses, such as bovine viral diarrhea viruses (BVDV), bovine respiratory syncytial virus (BRSV), parainfluenza-3 virus (PI3V) and bovine coronaviruses, and bacteria including *Mannheimia haemolytica, Pasteurella multocida, Histophilus somni* and *Mycoplasma* spp are the etiologies of life-threatening pneumonia known as bovine respiratory disease complex (BRDC), the most important disease in cattle [2–4]. BoHV-1 infection stimulates inflammasome formation [5], which contributes to BRDC by enhancing the inflammatory response in the lung. A BoHV-1 entry receptor, poliovirus receptor related 1, has been identified to be a BRDC susceptibility gene for Holstein calves [6], confirming the critical role of BoHV-1 as a cofactor for BRDC. BoHV-1 infection and the virus induced BRDC inflict a great economic lost to the cattle industry, worldwide [3].

It is known that BoHV-1 infection induces overexpression of pro-inflammatory cytokines, such as IL-1β and TNF-α that contribute greatly to the inflammatory response [7, 8]. In addition, we recently identified that BoHV-1 infection increases the generation of inflammatory mediator, reactive oxidative species (ROS) [9], which is also a potential mechanism to promote inflammatory response. Over-produced ROS mediated inflammatory response by diverse mechanisms in varied virus infections. In the context of Influenza virus or Dengue virus infection, ROS promotes the production of pro-inflammatory cytokines IL-1β and TNF-α [10–12]. In HSV-1 infected murine microglial cells, ROS mediated the regulation of some inflammation pertinent signaling, such as p38MAPK and Erk1/2 pathways [13]. In contrast, the activation of p38MAPK and Erk1/2 signaling by BoHV-1 infection is not mediated by over-produced ROS [14], though BoHV-1 and HSV-1 are genetically closed [9]. How BoHV-1 infection contributes to ROS production and the activation of p38MAPK and Erk1/2 signaling has yet to be found.

Phospholipase C (PLC) plays important roles in the regulation of inflammatory response with diverse mechanisms in varied cells, e.g. PLC signaling has been suggested to be involved in macrophage mediated inflammatory response by regulating ROS generation, inflammatory cytokine transcription, cell adhesion, and monocyte to macrophage differentiation [15–18]. NaCl-induced NLRP3 inflammasome activation in retinal pigment epithelial cells is partially dependent on the activities of PLC signaling [19]. The PLC family contains six members (β, γ, δ, ε, η and ζ) that are further subdivided into 13 isoforms, which are functionally dependent on the activation of a series of downstream signalings, such as protein kinase C (PKC) and calcium signaling [20]. It has been reported that PLCγ-1 signaling is required by influenza virus for efficient replication in both T-cells and human airway epithelial A549 cells, and for the virus induced inflammatory response mediated by macrophages [18, 21, 22], which highlighted the importance of PLC signaling in virus pathogenicity. However, the involvement of PLC signaling in BoHV-1 infection is unknown.

In this study, for the first time we investigated the role of PLC signaling in BoHV-1 infection. We demonstrated that BoHV-1 infection activated PLC signaling for efficient viral replication, increasing ROS generation and activation of Erk1/2 and p38MAPK signaling. These data collectively suggested that BoHV-1 infection regulated the generation of ROS and stimulation of Erk1/2 and p38MAPK signaling partially depending on PLC signaling. Our data suggest that PLC signaling is important for BoHV-1 infection and virus-induced inflammation.

## Materials and methods

### Antibodies and reagents

Antibodies against phospho-p38MAPK (Thr180/Tyr182), p38MAPK, phospho-p44/42 MAPK (Erk1/2) (Thr202/Tyr204), Erk1/2, phospho-PLCγ-1(Ser1248), PLCγ-1 and GAPDH, as well as HRP labeled secondary antibodies including goat anti-mouse IgG and goat anti-rabbit IgG were purchased from Cell Signaling Technology (Beverly, MA, USA). Fluorescein isothiocyanate (FITC) labeled goat anti-bovine IgG was purchased from Beijing Biosynthesis Biotechnology Co., Ltd (Beijing, China). PLC signaling inhibitors U73122 and edelfosine, intracellular ROS indicator 2′,7′- dichlorodihydrofluorescein diacetate (H2DCFDA), herpesvirus inhibitors phosphonoacetic acid (PAA) and Acyclovir (ACY), as well 3-(4,5-dimethylthiazol-2-yl)-2,5-diphenyl tetrazolium bromide (MTT) were purchased from Sigma-Aldrich (St. Louis, MO, USA).

### Cells and virus

MDBK cells and RS-1 cells were maintained in DMEM (Gibco BRL) supplemented with 10% horse serum and fetal bovine serum (HyClone Laboratories, Logan, UT, USA), respectively. BoHV-1 (Colorado1 stain) was propagated in MDBK cells. Aliquots of virus stocks were stored at −70 °C until use. The virus was titrated in MDBK cells with results expressed as TCID_50_/mL calculated using the Reed-Muench formula.

To inactivate BoHV-1 virus with UV-irradiation, virus stock was dispersed into 100-mm tissue culture dishes, and directly placed under a UV lamp (20 W) for 30 min. Complete inactivation of the virus was confirmed by the fact that no plaque was produced in MDBK cells exposed to the UV treated virus for 48 h.

### Cytotoxicity assays with MTT method

Cell viability was assessed by the MTT assay. MDBK or RS-1 cells were seeded into 96-well plates at 1 × 10^4^ cells/well within a volume of 200 μL per well. Eight replicates were mock treated with DMSO, U73122 or edelfosine at various concentrations of 5 and 10 μM for 24 h at 37 °C. Thirty microliters MTT solution (2 mg/mL in PBS) were added to each well. The cells were then incubated for 4 h at 37 °C. The medium was replaced with 150 μL of DMSO for each well to solubilize the formazan. The plates were shaken on a rotary platform for 10 min. Finally, the absorbance value was measured at a wavelength of 550 nm using a Wellscan (Labsystems, Santa Fe, NM, USA). The mean optical density of the control was assigned a value of 100%.

### Virus replication inhibition assay

Serum starved MDBK cell in 24-wells plates were pretreated with inhibitors at the designated concentration for 1 h at 37 °C, then infected with BoHV-1 (MOI of 1) for 1 h along with the treatment of corresponding inhibitors. After extensive washing with PBS, DMEM (400 μL) with or without inhibitors was added to each well. At 24 hpi, viral yields were titrated in MDBK cells. The results are expressed as TCID_50_/mL calculated using the Reed-Muench formula.

To test whether these inhibitors affected the viral entry or post entry stages of BoHV-1 infection, serum starved MDBK cells were incubated in 24-well plates with BoHV-1 (MOI = 1) for 1 h at 4 °C to allow the viruses to adsorb to the cell membrane but not to penetrate the cells. The cells were then subjected to extensive washing with ice-cold PBS. To identify the effect of PLC signaling on the virus entry process, pre-warmed fresh medium with DMSO, 5 μM of U73122, or 5 μM of edelfosine were added, and the cell cultures were quickly shifted to 37 °C for 1 h to allow the viruses enter the cells. The cells were then treated with citrate buffer (40 mM citric acid, 10 mM KCl, 135 mM NaCl, pH 3.0) for 1 min to inactivate cell membrane bound but unpenetrated virions [23, 24]. Subsequently, fresh medium was replaced and continuously incubated for 24 h at 37 °C. In parallel, to identify the effect of PLC signaling on the BoHV-1 post entry process, the virus adsorbed MDBK cells were first shifted to 37 °C for 1 h to allow the virus enter the cells followed by treatment with citrate buffer for 1 min, then incubated for 24 h at 37 °C in the absence or presence of U73122 and edlefosine, respectively. The virus yield was determined in MDBK cells with results expressed as TCID_50_/mL.

To test whether PLC signaling affects the virus binding to MDBK cells, serum starved MDBK cells were incubated in 6-well plates in the absence or presence of PLC inhibitors at a concentration of 5 μM for 1 h at 37 °C to block PLC signaling. Then the cells were incubated with virus stock at 4 °C for 1 h to allow the viruses absorb to the cell membrane, in the absence or presence of corresponding PLC inhibitors. Then the cells were washed with ice-cold PBS, and subjected to two rounds of frozen-thawings to release cell membrane attached viruses. After centrifugation, the viral titer was determined in MDBK cells, with data expressed as TCID_50_/mL.

### Flow cytometry assay

To analyze whether UV-inactivated BoHV-1 could bind to MDBK cells, the cells in 6-well plates were detached by treatment with 2 mM EDTA. After extensive washing with ice-cold PBS, the cells were incubated with infectious BoHV-1 or UV-inactivated BoHV-1 for 1 h at 4 °C allowing the viral particles to attach to the cells. The cells were then fixed with 4% paraformaldehyde for 15 min at room temperature, and stained with bovine anti-BoHV-1 serum followed by FITC-conjugated anti-bovine IgG. The cells were washed twice with ice-cold PBS, and the cell membrane bound viral particles were analyzed on FACS.

### Cellular ROS assay

MDBK cells in 24-well plates were pretreated with solvent DMSO, U73122 (2.5 μM) or edelfosine (5 μM) for 1 h, then infected with BoHV-1 (MOI = 10) in the presence of a corresponding inhibitor for 1 h. The uninfected control was treated with cell lysates from uninfected MDBK cells. After washing with PBS for three times, fresh medium containing inhibitor was added. At 4 hpi, the cells were washed with PBS and exposed to ROS fluorescence indicator H2DCFDA (50 μM) for 30 min at 37 °C. The reaction mixture was then replaced with PBS, and images were acquired under a fluorescence microscope, the fluorescence intensity of cellular ROS was quantified with software Image-pro Plus 6.

### Western blot analysis

To test the kinetic variation of PLCγ-1 signaling, monolayers of MDBK cells were serum starved overnight in 60-mm dishes, infected with BoHV-1(MOI = 10) for 0.5, 1, 2, 4, 8 and 12 h. Cell lysate was prepared using lysis buffer (1% Triton X-100, 50 mM sodium chloride, 1 mM EDTA, 1 mM EGTA, 20 mM sodium fluoride, 20 mM sodium pyrophosphate, 1 mM phenylmethylsulfonyl fluoride, 0.5 g/mL leupeptin, 1 mM benzamidine, and 1 mM sodium orthovanadate in 20 mM Tris–HCl, pH 8.0).

To test the effect of U73122 and edelfosine on the designated signaling, MDBK cells were serum starved overnight in 60-mm dishes, pretreated with U73122 or edelfosine at the indicated concentrations for 1 h, then mock-infected or infected with BoHV-1 at 37 °C for 0.5 h along with the treatment by the corresponding inhibitors. Cell lysates were then prepared with the lysis buffer as described above.

Cell lysate was separated on an 8 or 10% SDS–polyacrylamide gel and proteins were transferred to a polyvinylidene difluoride (PVDF) membrane (Bio-rad, CA, USA). After blocking with 5% nonfat milk in Tris-buffered saline (TBS) buffer containing 0.05% Tween 20 (TBST), the membrane was incubated with designated primary antibody (1:1000). After extensive washing with TBST, the HRP-conjugated secondary antibody (1:1500) in the blocking reagent was then added. After extensive washing with TBST, immune reactive bands were detected using the enhanced chemiluminescence (ECL) reaction substrate (Millipore, USA). The band intensity was analyzed with software image J.

## Results

### PLC signaling inhibitors U73122 and edelfosine inhibited BoHV-1 infection in bovine kidney cells (MDBK cells) and rabbit skin cells (RS-1 cells)

**Figure 1:**
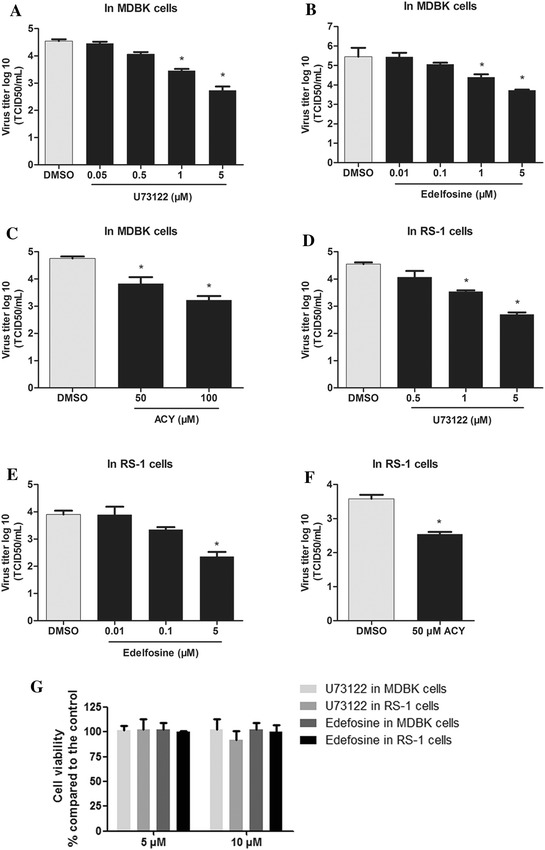
**Inhibitory effect of U73122 and edelfosine on BoHV-1 viral replication in vitro.** Inhibitory effect of U73122 (**A**), edelfosine (**B**) and ACY (**C**) on BoHV-1 replication in MDBK cells. Serum starved MDBK cells were pretreated with each inhibitor at indicated concentrations for 1 h, infected with BoHV-1(MOI = 1) for 24 h along with the treatment of corresponding inhibitor, respectively. The viral titer was determined with MDBK cells with the results expressed as TCID_50_/mL. Inhibitory effect of U73122 (**D**), edelfosine (**E**) and ACY (**F**) on BoHV-1 replication in RS-1 cells. Serum starved RS-1 cells were pretreated with each inhibitor at the indicated concentrations for 1 h, infected with BoHV-1(MOI = 1) for 24 h along with the treatment of the corresponding inhibitor, respectively. The viral titer was determined with MDBK cells with results expressed as TCID_50_/mL. (**G**) The cytotoxicity assay for both U73122 and edelfosine in either MDBK cells or RS-1 cells. MDBK cells or RS-1 cells were exposed DMSO, U73122 or edelfosine at the designated concentrations for 24 h. After incubation with MTT, the OD value was determined and compared to the control which was designated as 100%. Data represent three independent experiments. Significance was assessed with the student *t* test (*P < 0.05).

To test whether PLC signaling is involved in BoHV-1 infection, the effect of chemical inhibition of PLC signaling on viral replication in MDBK cells was first investigated. Since the chemical inhibitors may have an off target effect, we used two PLC signaling specific inhibitors U73122 and edelfosine for this evaluation in parallel. As shown in Figures 1A and B, the treatment of MDBK cells with U73122 or edelfosine resulted in a reduction of the virus yield in a dose-dependent manner. With the treatment of 1 and 5 μM of both inhibitors, the virus yield reduced a titer of ~1 and 2-log in comparison with the control samples, respectively. Acyclovir (ACY) a known inhibitor for herpesvirus was introduced as a positive control. ACY at a concentration of 50 and 100 μM decreased the virus titer by ~0.9 and 1.5-log compared to the control, respectively (Figure 1C), validating the inhibitory effects of both U73122 and edelfosine on BoHV-1 replication in MDBK cells. These results suggest that PLC signaling is required for BoHV-1 infection in MDBK cells. Rabbit skin (RS-1) cells are also permissive for BoHV-1 productive infection [25]. To see whether the inhibitory effect of PLC inhibitors on BoHV-1 replication was bovine kidney cell specific, RS-1 cells were utilized for further evaluation. As can be seen in Figure 1D, E, both inhibitors at a concentration of 5 μM significantly reduced the virus yield in RS-1 cells. The known herpesvirus inhibitor ACY at a concentration of 50 μM decreased the virus titer of ~1-log compared to the control (Figure 1F), which validated the inhibitory effects of both U73122 and edelfosine on BoHV-1 replication in RS-1 cells. In addition, the reduced virus yield by both U73122 and edelfosine in both MDBK cells and RS-1 cells was not due to the chemical cytotoxicity, because both inhibitors at the tested concentrations did not show apparent cytotoxicity to neither MDBK cells nor RS-1 cells detected with the MTT assay (Figure 1G). Therefore, our data suggest that PLC signaling may affect BoHV-1 infection in a broad spectrum of cell types, in vitro.

### PLC signaling inhibitors U73122 and edelfosine inhibited BoHV-1 infection at the postbinding cell entry stages

**Figure 2:**
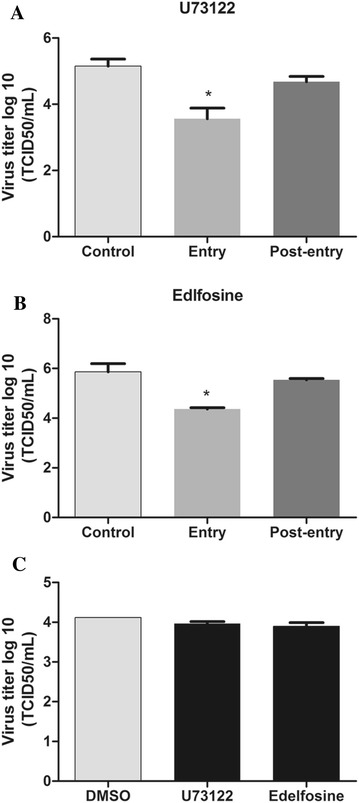
**Both U73122 and edelfosine affect virus entry.** Serum starved MDBK cells infected with BoHV-1 (MOI = 1) were treated with U73122 (**A**) and edelfosine (**B**) at a concentration of 5 μM, at the virus entry, or post-entry process, respectively. At 24 hpi the viral titer were determined in MDBK cells with results expressed as TCID50/mL. **C** Serum starved MDBK cells were incubated in the absence or presence of 5 μM of U73122 or 5 μM of edelfosine for 1 h at 37 °C. The virus in ice-cold DMEM containing fresh inhibitors was then allowed to adsorb to MDBK cells for 1 h at 4 °C. After freezing and thawing, the cell membrane bound viruses were titered in MDBK cells. Values represent three independent experiments. Significance was assessed with the student *t* test (*P < 0.05).

Since PLC inhibitor of both U73122 and edelfosine could unanimously inhibit BoHV-1 replication in both MDBK and RS-1 cells, we further identified which stage(s) of the viral replication including virus binding, postbinding cell entry and post entry stages was affected in MDBK cells. As a result, both U73122 and edelfosine mainly affected the virus postbinding cell entry stages but not the post entry stages (Figures 2A and B). As virus attachment and cell entry are early events in the virus infection cycle, we further investigated the role of PLC signaling in virus attachment. As shown in Figure 2C, both U73122 and edelfosine treatment had no effect on BoHV-1 binding to the cell surface. These results suggest that PLC signaling may mainly contribute to the postbinding cell entry stages during BoHV-1 infection of MDBK cells.

### BoHV-1 infection activated PLCγ-1 signaling in MDBK cells

**Figure 3:**
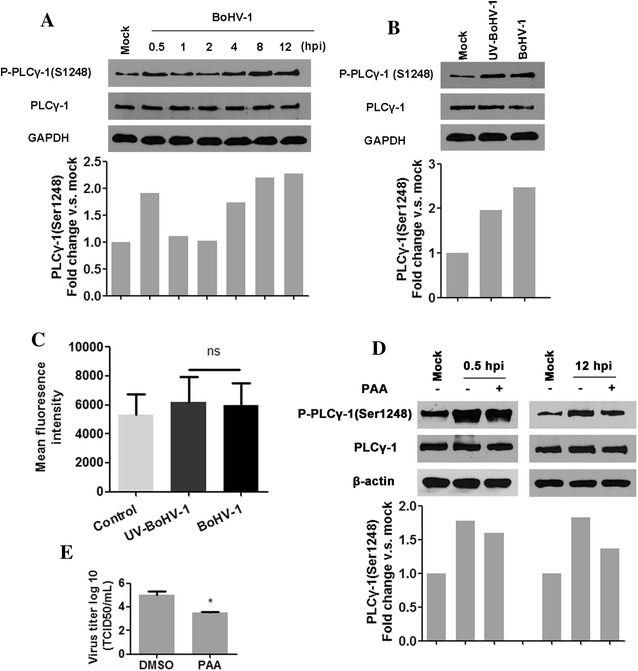
**BoHV-1 infection enhanced the phosphorylation of PLCγ-1 (S1248) in MDBK cells. (A)** Time course of PLCγ-1(Ser1248) phosphorylation following BoHV-1 infection. Serum starved MDBK cells were infected with BoHV-1 at an MOI of 10, and at indicated time points the cell lysates were prepared for western blotting. (**B**) MDBK cells were incubated with UV-irradiation inactivated virus or infectious virus at an MOI of 10 for 30 min, and then subjected to analysis of PLCγ-1(Ser1248) phosphorylation by western blotting. (**C**) The ability of UV-irradiation inactivated virus to bind to MDBK cells were analyzed with FACS. MDBK cells with or without viral particles attached were stained with bovine anti-BoHV-1 serum followed by FITC-conjugated anti-bovine IgG. The cell membrane bound viral particles were analyzed on FACS. The mean fluorescence intensity was analyzed. (**D**) Serum starved MDBK cells were infected with BoHV-1 at an MOI of 10 along with PAA treatment (100 μM). At 0.5 and 12 hpi the cell lysates for western blotting analysis were prepared, respectively. At 24 hpi the virus yield were determined in MDBK cells (**E**). The band intensity was analyzed with software image J. Data are representative results of three independent experiments.

Since PLC signaling inhibitor apparently affected BoHV-1 replication in both MDBK and RS-1 cells, we further examined whether BoHV-1 infection affected PLC signaling by testing the kinetics of phosphorylated PLCγ-1(Ser1248) during virus infection of MDBK cells as previously described [18, 21]. In order to see the burst effect of virus infection on the detected signaling, MDBK cells were infected with BoHV-1 (Colorado 1 strain) at a high MOI of 10. At 0.5, 1, 2, 4, 8 and 12 hpi, the cell lysate were prepared and subjected to western blot analysis. As shown in Figure 3A, the level of phospho-PLCγ-1(Ser1248) was first detected to increase at 0.5 hpi, then it decreased to the basal level from 1 to 2 hpi, and re-increased at 4 hpi, then remained at a high level until the end of the detected time point at 12 hpi.

Since the increased phosphorylation of PLCγ-1(Ser1248) could be detected at 0.5 hpi, we wondered whether this was related to viral entry. To address this question, the ability of UV-inactivated BoHV-1 virus to stimulate the phosphorylation of PLCγ-1(Ser1248) in MDBK cells was examined as previously described [21]. UV-inactivated virus could bind to the virus receptors and enter the cells, but could not express viral genes [26]. Here, the ability for UV-irradiated viral particles to bind to MDBK cells was confirmed with FACS assay (Figure 3C). As expected, UV-irradiated BoHV-1 could also increase the phosphorylation of PLCγ-1(Ser1248), compared to the control, though there is a minor reduced ability compared to the infectious viral particles (Figure 3B). This suggests that the virus entry would initially stimulate the phosphorylation of PLCγ-1(Ser1248) which corroborated the result that PLC signaling inhibitors affected the virus entry stage(s) (Figure 2A). We also supposed that de novo viral protein expression or DNA replication may potentially account for the enhanced phosphorylation of PLCγ-1(Ser1248) that occurred at 4, 8 and 12 hpi (Figure 3A). To test the hypothesis, MDBK cells were treated with phosphonoacetic acid (PAA), a specific inhibitor targeting the viral DNA polymerase [27], through virus infection. As a result, PAA treatment significantly inhibited the virus replication in MDBK cells (Figure 3E), and apparently reduced the phosphorylation of PLCγ-1(Ser1248) at 12 hpi but not at 0.5 hpi which was stimulated by BoHV-1 infection while PAA treatment only had a minor effect on viral induced phosphorylation of PLCγ-1(Ser1248) at 0.5 hpi (Figure 3E). Thus, de novo viral protein production and/or DNA replication seems to be correlated to the elevated levels of phosphorylated PLCγ-1(Ser1248).

### PLC signaling inhibitors U73122 and edelfosine inhibited Erk1/2 and p38MAPK signaling stimulated by BoHV-1 infection

**Figure 4:**
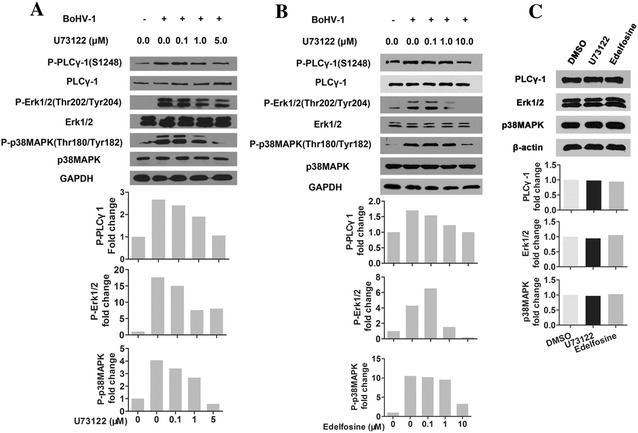
**Effect of U73122 and edelfosine on Erk1/2 and p38MAPK signaling in response to BoHV-1 infection.** Serum starved MDBK cells in 60-mm dishes were pretreated with U73122 (**A**) or edelfosine (**B**) at the indicated concentrations for 1 h, respectively, then infected with BoHV-1 (MOI = 10) in the presence of respective inhibitors for 0.5 h. (**C**) Serum starved MDBK cells in 60-mm dishes were exposed to DMSO, U73122 or edelfosine at the indicated concentrations for 1.5 h. Cell lysate was prepared and subjected to western blotting analysis. The band intensity was analyzed with software image J. The data are representatives of three independent experiments.

As we know, the activated PLC signaling can stimulate calcium and PKC signaling [33]. In addition, multiple studies have demonstrated that Erk1/2 and p38MAPK signaling can be activated by PKC and increased intracellular calcium signaling [28, 29], suggesting that BoHV-1 may activate Erk1/2 and p38MAPK signaling through PLC signaling. Therefore, the effects of U73122 and edelfosine on Erk1/2 and p38MAPK signaling in response to BoHV-1 infection were examined. As expected, both inhibitors decreased the levels of phosphorylated PLCγ-1(Ser1248) in a dose-dependent manner (Figures 4A and B; P-PLCγ-1 panel), validating the specific effect of these chemicals on PLC signaling. BoHV-1-induced activation of Erk1/2 and p38MAPK, was attenuated by U73122 and edelfosine in a dose-dependent manner (Figures 4A and B, P-Erk1/2 and P-p38MAPK panels). However, these inhibitor treatments did not have a dramatic effect on total steady state protein levels of PLCγ-1, Erk1/1 or p38MAPK following BoHV-1 infection (Figure 4C). Therefore, our results indicated that the activation of Erk1/2 and p38MAPK signaling by BoHV-1-infection was partially dependent on PLC signaling.

### PLC signaling inhibitors U73122 and edelfosine inhibited ROS generation stimulated by BoHV-1 infection

**Figure 5:**
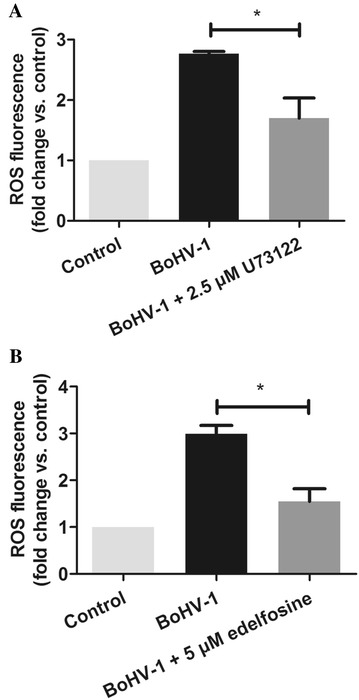
**The reduction of ROS levels by PLC inhibitor U73122 in MDBK cells following BoHV-1 infection.** MDBK cells subjected to a pretreatment with DMSO, U73122 (2.5 μM) (**A**) or edelfosine (5 μM) (**B**) for 1 h, were mock infected with supernatant of cell culture or infected with BoHV-1 along with chemical inhibitors. At 4 hpi cellular ROS were detected using H2DCFDA (5 μM, 30 min), and the quantification of fluorescence intensity was analyzed using software Image-pro Plus 6. Values represent three independent experiments. Significance was assessed with the student *t* test (*P < 0.05).

We recently reported that BoHV-1 infection promotes ROS production which depended on viral entry, and de novo protein expression and/or DNA replication [9]. Here, we additionally tested whether chemical inhibition of PLC signaling affected BoHV-1-induced ROS production. Our results indicate that the treatment of MDBK cells with PLC inhibitor of both U73122 (2.5 μM) and edelfosine (5 μM) led to decreased ROS production stimulated by BoHV-1 infection by 38.6 and 48.3%, respectively (Figure 5). It indicated that PLCγ-1 signaling may partially regulate ROS production in response to BoHV-1 infection.

## Discussion

In this study, for the first time we demonstrated that chemical inhibition of PLC signaling led to significant inhibition of BoHV-1 infection. Since chemical inhibitors may have off target effects, two specific inhibitors for PLC signaling U73122 and edelfosine were employed, in parallel, with both bovine kidney cells and rabbit skin cells. They unanimously show inhibitory effects on BoHV-1 infection in both cell cultures (Figure 1). Biphasic activation of PLCγ-1 signaling by BoHV-1 infection in MDBK cells corroborated the requirement of PLC signaling in the virus infection (Figure 3).

Since PLC signaling is potentially regulating intracellular calcium signaling, multiple evidence suggests that calcium signaling is important to mediate some virus entry processes, e.g., Ca^2+^ is strictly required for the cell entry of Rubella virus [30], Ebola virus, Marburg virus, Lassa virus and Junin virus [31]. In this study, our findings suggest that PLC inhibitors of both U73122 and edelfosine could efficiently inhibit the virus postbinding cell entry process (Figure 2). It is possible that PLC signaling mediated BoHV-1 entry through the mobilization of calcium signaling, which needs further investigation.

We recently reported that BoHV-1 infection increased ROS production, which was required for efficient viral replication, and the oxidative stress contributes to mitochondrial dysfunction in MDBK cells [9]. However, the mechanism underlying the overproduction of ROS driven by BoHV-1 infection is still unknown. Here, we illustrated that inhibition of PLC signaling led to a significant decrease of ROS production induced by BoHV-1 infection (Figure 5), indicating that the virus induced ROS generation was partially regulated by PLC signaling.

It is known that the NADPH (nicotinamide adenine dinucleotide phosphate) oxidases (NOXs) family including NOX1- to −5 and Duox1- to −2 are the main resource of ROS generation [32]. We previously identified that PLC signaling inhibitor U73122 inhibits ROS production partially through decreasing NOX2 expression in influenza virus PR8-infected human macrophage like cells (dU937 cells) [18]. However, antibodies suited for the detection of bovine NOXs are currently unavailable, and we could not identify whether NOXs expression is regulated by PLC signaling in the context of BoHV-1 infection. However, we know that BoHV-1 induced ROS production is partially regulated by PLC signaling.

Accordingly and in view of the extensive published evidence regarding the interaction between calcium signaling and MAPK pathways, as well as the mobilization of calcium signaling by PLC signaling [33], we further identified the effect of PLC signaling on Erk1/2 and p38MAPK pathways. Here, we show that inhibition of PLC signaling attenuated the activation of Erk1/2 and p38MAPK signaling following BoHV-1 infection, but did not affect the steady state levels of both Erk1/2 and p38MAPK (Figure 3), indicating that PLC signaling partially mediates the activation of Erk1/2 and p38MAPK signaling stimulated by BoHV-1 infection. It is highly possible that BoHV-1 infection activates this MAPK signaling via the PLC/calcium signaling axis.

In summary, in this study we describe a so far unrecognized role of PLC signaling in BoHV-1 infection. For the first time we identified that PLC signaling is not only important for the BoHV-1 infection, but also important for the activation of Erk1/2 and p38MAPK signaling as well as excessive production of ROS induced by the virus infection. The activation of PLC signaling is a potential pathogenic mechanism for BoHV-1 infection. Therefore, pharmacological modulation of PLC signaling may provide a novel approach for fighting the virus through inhibition of both viral replication and inflammatory response.
